# Colloidal Synthesis of NbS_2_ Nanosheets: From Large-Area Ultrathin Nanosheets to Hierarchical Structures

**DOI:** 10.3389/fchem.2020.00189

**Published:** 2020-04-07

**Authors:** Wenhui Li, Xijun Wei, Hongmei Dong, Yingqing Ou, Shenghuan Xiao, Yang Yang, Peng Xiao, Yunhuai Zhang

**Affiliations:** ^1^College of Chemistry and Chemical Engineering, Chongqing University, Chongqing, China; ^2^College of Physics, Chongqing University, Chongqing, China

**Keywords:** colloidal synthesis, morphology regulation, niobium disulfide nanosheets, supercapacitor, transition metal dichalcogenides

## Abstract

Layered NbS_2_, a member of group-V transition metal dichalcogenides, was synthesized via a colloidal synthesis method and employed as a negative material for a supercapacitor. The morphologies of NbS_2_ can be tuned from ultrathin nanosheets to hierarchical structures through dynamics controls based on growth mechanisms. Electrochemical energy storage measurements present that the ultrathin NbS_2_ electrode exhibits the highest rate capability due to having the largest electrochemical surface area and its efficient ion diffusion. Meanwhile, the hierarchical NbS_2_ shows the highest specific capacitance at low current densities for small charge transfer resistance, displays 221.4 F g^−1^ at 1 A g^−1^ and 117.1 F g^−1^ at 10 A g^−1^, and cycling stability with 78.9% of the initial specific capacitance after 10,000 cycles. The aggregate or stacking of nanosheets can be suppressed effectively by constructing hierarchical structure NbS_2_ nanosheets.

## Introduction

Layered transition-metal dichalcogenides (TMDs), which have a X-M-X sandwich structure (generalized formula: MX_2_, M = transition metal element; X = S, Se, or Te) in each layer, have attracted extensive attention due to their great potential for applications in energy storage (Chhowalla et al., 2013; Muller et al., 2015; Lin et al., 2019), catalysis (Yan et al., 2014; Yang et al., 2019), electronics (Wang et al., 2012; Lin et al., 2018), photonics (Mak and Shan, 2016; Linhart et al., 2019) etc. Recently, significant progress has been made on TMDs materials for energy storage applications (Han et al., 2018; Yun et al., 2020). However, a crucial issue regarding practical use is the aggregation or restacking of the individual layers with high surface energy by interlayer van der Waals forces (Chhowalla et al., 2013), which can cause low coulombic efficiency and capacity degradation irreversibly (Mei et al., 2018).

To solve the aggregation or restacking problem, some strategies to optimize the structure of TMDs materials have been proposed in previous studies. One strategy is to construct TMDs/conductor composites, e.g., grapheme (Li et al., 2011; Yang et al., 2017), carbon nanotubes (Wang et al., 2019), carbon nanofibers (Cha et al., 2017), organic polymers (Cho et al., 2019), etc., which can suppress the aggregation of layered TMDs effectively. Another important strategy is to adjust the structure parameters in the synthesis process. For instance, to tune the size and thickness of nanosheets (Yin and Alivisatos, 2005; Yoo et al., 2014; Mansouri and Semagina, 2018), increase the layer-to-layer spacing (Jang et al., 2011), or construct hierarchical architectures (Sun et al., 2014; Cong et al., 2017; Zhang J. et al., 2017).

Among layered TMDs, group-V 2H-NbS_2_ has an intrinsic metallic character owing to the half-filled 4$d_{z^{2}}$orbital (Kuc et al., 2011). The good electric conductivity and preferable flexibility of 2H-NbS_2_ are practical for applications in energy storage (Han et al., 2018). However, the control of the morphologies, growth mechanism, and the structure-property relationship still need to be further explored. Therefore, this paper regulated the reaction dynamics of the colloidal synthesis process of 2H-NbS_2_ by adjusting growth temperature and the amount of carbon disulfide, obtaining different morphologies of NbS_2_, such as ultrathin nanosheets, self-assembled hierarchical structures, and layer stacked nanosheets. Furthermore, the growth mechanism was illustrated, and the effect of morphologies on supercapacitor performance was investigated.

## Experimental Section

### Chemicals

Niobium (V) chloride (NbCl_5_, 99.9%), carbon disulfide (CS_2_, 99.9%), and oleylamine (80–90%) were purchased from Aladdin Ltd. Potassium chloride (KCl), Hexane, n-butanol, and absolute ethanol were of analytical grade and all obtained from Chuan Dong Ltd.

### Synthesis of NbS_2_ Nanosheets

A slight modified colloidal synthesis method is used to synthesize NbS_2_ nanosheets (Jeong et al., 2012). First, NbCl_5_ (1 mmol, 0.27 g) and oleylamine (24.3 mmol, 8.0 mL) was added to a 100 mL three-neck flask under Ar atmosphere. Next, the mixture was treated under ultrasound until completely dissolved of NbCl_5_ to form yellow transparent liquid. Then the mixture was heated to 120°C for 30 min under an Ar flow to remove water and oxygen. After the solution was heated to 300°C, CS_2_ (30 mmol, 1.8 mL) was injected dropwise into the hot solution at a speed of ~1 mL/min. The reaction proceeded at 300°C for 2 h before being stopped by removal from the heating mantle. The NbS_2_ nanosheets were precipitated by addition of excess butanol, and washed by mixed solution of hexane and absolute ethanol (1:1), and DI water for several times. Finally, the brownish black NbS_2_ powder was obtained by a freeze-drying method. To regulate the morphologies of NbS_2_, the reaction temperature was set at 280, 300, and 320°C, and the CS_2_ amount was adjusted as 10, 30, and 60 mmol, respectively, and other experiment parameters remained unchanged.

### Material Characterization

Scanning electron microscopy (SEM) images were taken on a field-emission scanning electron microscope (FESEM, FEI Nova 400 Nano-SEM). A high-resolution transmission electron microscope (HRTEM, ThermoFisher Scientific, Talos F200s) was performed to analyze the structures and elemental mapping. X-ray diffraction (XRD) patterns of the as-synthesized NbS_2_ were recorded on a diffractometer with Cu Kα radiation (Spectris Pte. Ltd, PANalytical X' Pert Powder). Chemical states of the containing elements were analyzed by X-ray photoelectron spectroscopy (XPS, Thermo Scientific ESCALAB 250Xi, K-alpha). Fourier transform infrared (FTIR) spectrometer (ThermoFisher Scientific, Nicolet iS50) was used to analyze the composition of the samples.

### Electrochemical Measurement

The electrochemical performance of NbS_2_ nanosheets for the supercapacitor was measured by using a three-electrode cell system at room temperature. Specifically, the active material (70 wt%), conductive carbon black (20 wt%), and polyvinylidene fluoride binder (10 wt%) were mixed and turned into slurry uniformly, using absolute ethanol. Then the slurry was coated onto the pre-cleaned nickel foam with mass loading of active material ~1.5 mg cm^−2^. After being dried in a vacuum oven at 60 °C overnight, the nickel foam coated with active material and another pre-cleaned nickel foam were overlapped and pressed at 10 MPa and used as a working electrode. A platinum foil and an Ag/AgCl (saturated KCl) electrode were used as the counter and reference electrode, respectively. The electrochemical performance was tested in 1 M KCl electrolyte solutions. Cyclic voltammetry (CV), galvanostatic charge-discharge (GCD) curves and electrochemical impedance spectroscopy (EIS) were tested using an electrochemical workstation (CHI660E, CH Instruments). Cycling stability tests were carried out on an electrochemical instrument (LAND).

## Results and Discussion

### Physical Characterization of NbS_2_ Nanosheets

**Figure 1:**
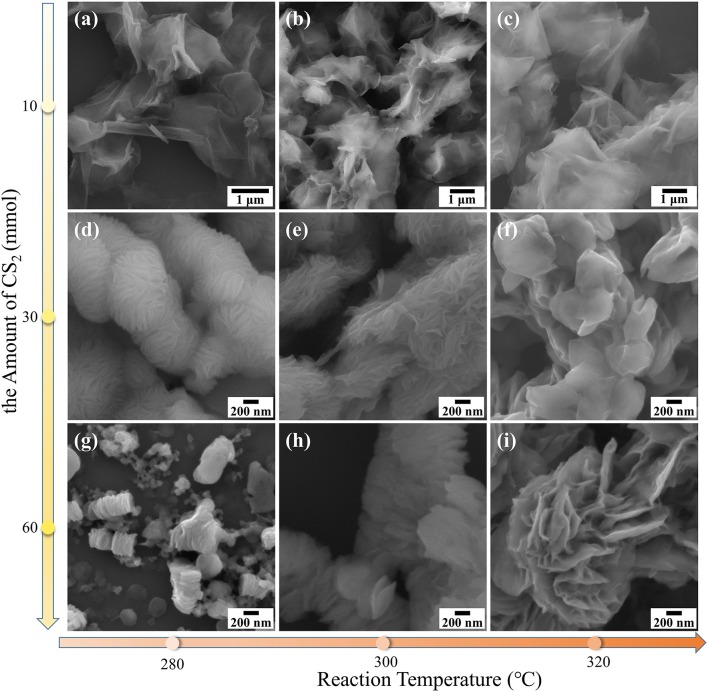
SEM images of NbS_2_ nanosheets synthesized at different reaction temperatures and carbon disulfide (CS_2_) amounts at high magnification. **(a)** 280°C, 10 mmol CS_2_. **(b)** 300°C, 10 mmol CS_2_. **(c)** 320°C, 10 mmol CS_2_. **(d)** 280°C, 30 mmol CS_2_. **(e)** 300°C, 30 mmol CS_2_. **(f)** 320°C, 30 mmol CS_2_. **(g)** 280°C, 60 mmol CS_2_. **(h)** 300°C, 60 mmol CS_2_. **(i)** 320°C, 60 mmol CS_2_.

The morphologies of NbS_2_ nanosheets can be tuned by adjusting the reaction conditions. The lateral size, thickness, and assembly mode of NbS_2_ nanosheets varied with the reaction temperature and the amount of carbon disulfide (CS_2_), as observed by SEM images [Figure 1 (high magnification), Figure S1 (low magnification)]. The materials exhibited large lateral size and ultrathin nanosheets when using 10 mmol CS_2_ as the sulfur source at a reaction temperature of 280, 300, and 320°C (Figures 1a–c, Figures S1a–c). The lateral size of the nanosheets was ~2 μm at 280°C, ~3 μm at 300°C, and ~4 μm at 320°C, and showed an increasing trend with reaction temperature. Scrolled structures could be observed at the edges of the ultrathin films. The nanosheets formed unavoidable wrinkles as a self-folding phenomenon to balance adhesion energy and bending strain energy, which can cause the intrinsic lattice structures to distort (Chen et al., 2019).

With the increase of the amount of CS_2_, the NbS_2_ nanosheets tended to assemble to form hierarchical or stacked structures, including both vertically assembled (face-to- face) and laterally gathered (edge-to-edge) ways (Zhang X. et al., 2017), and the lateral size showed a decreasing trend while the thickness increased. Under the reaction condition of 300°C and 30 mmol CS_2_, the materials grew to hierarchical structures, composed of nanosheets with lateral sizes of ~700 nm (hierarchical NbS_2_, Figure 1e, Figure S1e). When the amount of CS_2_ was increased to 60 mmol at 300 °C, the NbS_2_ structures was assembled by layer-stacked nanodisks, which lateral size reduced to ~500 nm (stacked NbS_2_, Figure 1h, Figure S1h). At 280 °C and 60 mmol CS_2_ conditions, the nanodisks with diameters of ~300 nm were mainly vertically assembled, like granum that stack thylakoids (Figure 1g, Figure S1g). At 320°C and 60 mmol CS_2_ conditions, the nanosheets with diameters of ~1 μm formed flower-like structures (Figure 1i, Figure S1i). Overall, the lateral size and thickness of NbS_2_ nanosheets increased with the temperature. With the increasing amount of CS_2_, the lateral size decreased and the thickness increased.

To simplify the study, the ultrathin, hierarchical, and stacked NbS_2_ nanosheets in the following test results of this essay refer to the samples synthesized using 10, 30, and 60 mmol CS_2_ at 300°C, corresponding to Figures 1b,e,h respectively.

**Figure 2:**
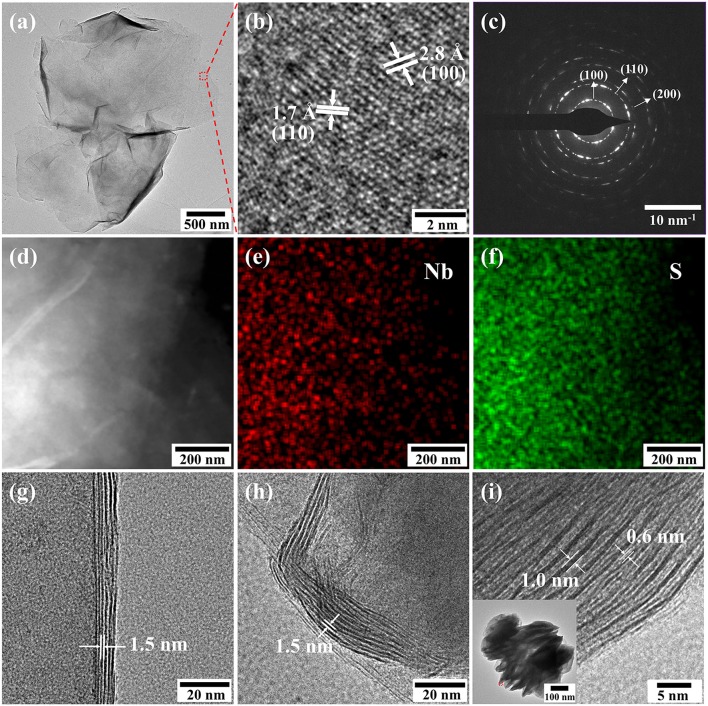
**(a)** TEM images, **(b)** HRTEM images and **(c)** SAED pattern of ultrathin NbS_2_ nanosheets. **(d–f)** Element mapping of ultrathin NbS_2_ nanosheets. **(g–i)** Edges of ultrathin, hierarchical and stacked NbS_2_ nanosheets. Inset: TEM images of stacked NbS_2_ nanosheets.

The TEM images of the NbS_2_ nanosheets are shown in Figure 2. The lateral size of the ultrathin NbS_2_ nanosheets was about 3 μm (Figure 2a). The lattice fringes of the ultrathin nanosheet presented in HRTEM images indicate the as-prepared NbS_2_ was in 2H phase (Figure 2b). The interplanar spacings of 2.8 and 1.7 Å corresponded to the (100) and (110) plane of 2H-NbS_2_ (Jeong et al., 2012). The diffraction rings in the selected area electron diffraction (SAED) pattern illustrated a polycrystalline nature, which can be assigned to (100), (110), and (200) planes, respectively (Figure 2c). The element mapping of the scanning transmission electron microscope-energy dispersive spectroscope (STEM-EDS) presented the uniform distributions of Nb and S in the nanosheets (Figures 2d–f). Figures 2g–i show the edges of ultrathin, hierarchical, and stacked nanosheets, corresponding to Figures 1b,e,h, respectively. The enlarged layer-to-layer spacings of ultrathin and hierarchical nanosheets were ~1.5 nm, indicating the existence of OLA molecules among layers (Jang et al., 2011). The 0.6 nm spacing of stacked nanosheets was consistent with the (002) lattice plane, while the 1.0 nm spacing could also be expanded by OLA molecules. The inset pattern in Figure 2i shows the TEM images of stacked NbS_2_ nanosheets. The thickness of the NbS_2_ nanosheets increased with the amount of CS_2_, from 10, 20, to 60 nm.

**Figure 3:**
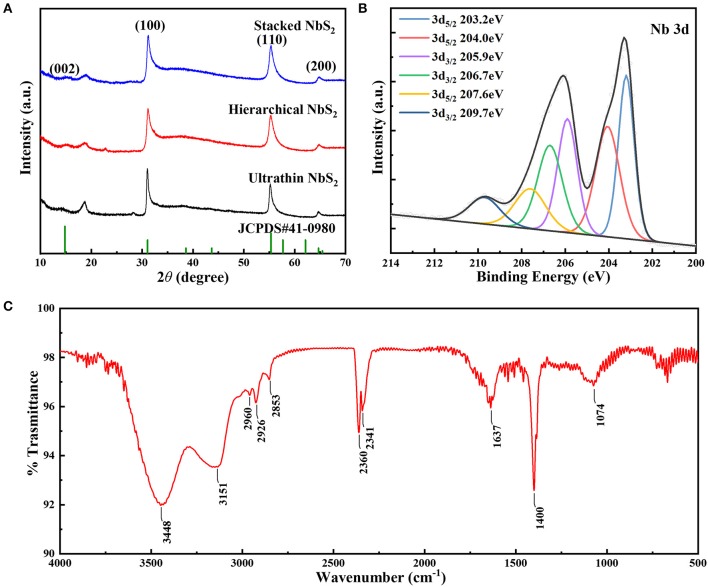
**(A)** XRD patterns of ultrathin, hierarchical and stacked NbS_2_ nanosheets (NbS_2_, JCPDS no.41-0980). **(B)** XPS results of Nb 3d and **(C)** FT-IR spectra of the hierarchical NbS_2_ nanosheets.

The crystalline characteristics of the various NbS_2_ samples with different morphologies were confirmed by XRD, as shown in Figure 3A. The diffraction peaks of all the as-prepared NbS_2_ matched with the standard pattern (JCPDS no.41-0980), the peaks at 14.8°, 31.0°, 55.3° and 64.7° could be assigned to (002), (100), (110), and (200) planes of the hexagonal crystal structure NbS_2_ with space group *P*63/*mmc* (Zhang J. et al., 2017), which was consistent with the lattice fringes of the HRTEM results.

To analyze the surface electronic states of the NbS_2_ nanosheets, an XPS survey was carried out. Figure 3B shows the Nb 3d spectrum of the hierarchical NbS_2_ nanosheets. The binding energies of 3d_3/2_ located at 209.7 eV and 3d_5/2_ at 207.6 eV revealed the existence of Nb^5+^ (Zhang J. et al., 2017). The peaks of 3d_3/2_ at 206.7 eV and 3d_5/2_ at 204.0 eV could be assigned to Nb^4+^ (Zhang J. et al., 2017). The binding energies of 3d_3/2_ state occurring at 205.9 eV and 3d_5/2_ at 203.2 eV corresponded to the oxidation state of Nb^(4−δ)+^ (Izawa et al., 2008). The Nb 3d and S 2p XPS spectra of ultrathin, hierarchical and stacked NbS_2_ nanosheets were summarized in Figure S2. In the S 2p spectrum (Figure S2b), peaks of 2p_2/3_ and 2p_1/2_ at 160.6 and 161.7 eV respectively, was corresponding to S^2−^ (Dash et al., 2015).

To examine the surfactant on the surface of the samples, an FT-IR test of hierarchical NbS_2_ nanosheets ranging from 500 ~ 4,000 cm^−1^ was conducted (Figure 3C). The FT-IR spectra proved the existence of oleylamine (OLA). The two broad absorption peaks at 3448 and 3151 cm^−1^ could be assigned to N–H stretching mode, and peak at 1637 cm^−1^ corresponded to NH_2_ scissoring mode (Altavilla et al., 2011; Cooper et al., 2011). The peaks at 2,960 and 2,926 cm^−1^ were due to asymmetrical stretching vibration of CH_3_, and the peak at 2,853 cm^−1^ was due to asymmetrical stretching vibration of CH_2_ (Altavilla et al., 2011). The broad peak located at 1074 cm^−1^ came from C–N stretching vibration mode (Gunasekaran et al., 2008). The peaks at 2,360 and 2,341 cm^−1^ were attributed to the adsorption doublet band of CO_2_. The peak located at 1,400 cm^−1^ could be due to CH_3_ symmetric bending vibration (Gunasekaran et al., 2008).

### Growth Mechanism of NbS_2_ Nanosheets

**Figure 4:**
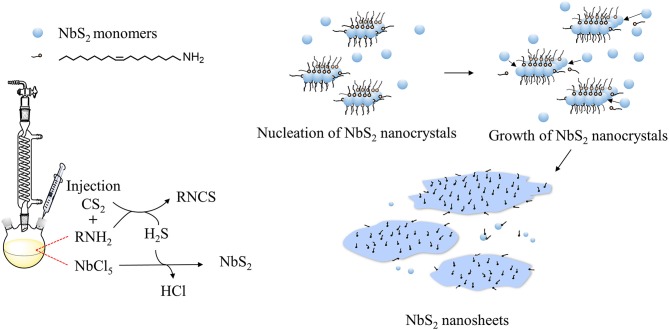
Growth mechanism of NbS_2_ nanosheets.

The shape of nanocrystals is dominated by the surface energy of the facets. For some transition metal dichalcogenides, including NbS_2_, 2D shapes are thermodynamically favored (Jeong et al., 2012; Moon, 2018). In the synthesis process of colloidal NbS_2_ nanosheets, oleylamine (OLA) serves as an organic surfactant, solvent, and reducing agent, as reported in other colloidal synthesis works (Yin and Alivisatos, 2005). The reaction can be illustrated as the following steps: (1) NbCl_5_ and OLA formed complexes by the dissolution of NbCl_5_ in OLA (Nasilowski et al., 2016); (2) generation of H_2_S gas via the reaction of OLA with CS_2_ after the injection of CS_2_ (Yoo et al., 2014); (3) reaction of the niobium (V) with H_2_S to form Niobium (IV) disulfide monomers; (4) burst of nucleation of NbS_2_ crystals; (5) growth of NbS_2_ crystals to form nanosheets (Figure 4).

Temperature is an important factor for crystal growth as it can change dynamics of ions and atoms in solution, and also alter the probability of effective collision between atoms. The adhesion of OLA molecules as surfactant to the NbS_2_ nanocrystal surfaces can form NbS_2_ – OLA complexes, and the stability and motion rate can be adjusted by the growth temperature. The OLA molecules should be capable of exchanging on and off the surface of growing crystals for instantaneous growth (Yin and Alivisatos, 2005). As the temperature rises, the stability of the complexes reduces, and OLA molecules have a higher probability of leaving the NbS_2_ nanocrystals, then monomers are more likely to access and add to the nanocrystal surface—thus the thickness and lateral size of the nanosheets increase, which is consistent with the experiment results.

The morphology changes, including lateral size and thickness, caused by the dose of CS_2_ can be explained by the following two reasons. On one hand, the amount of CS_2_ injected into the reaction system may change the monomer concentration. According to “size-distribution focusing” theory (Yin and Alivisatos, 2005), the critical size of nanocrystals diminishes instantly after injection of precursor for a second time due to the burst of monomers. In the synthesis process of NbS_2_ nanocrystals in our experiment, CS_2_ was injected slowly (about 0.1 mL/min), so the higher amount of CS_2_, the longer the injection time is. Based on the condition that an excess of OLA acts as a reducing agent, and the quick enough reduction of CS_2_ by OLA, as well as the rapid reaction between NbCl_5_ and H_2_S, it is hypothesized that the generation of monomers occur instantly and continuously during the CS_2_ injection period, and the critical size of NbS_2_ nanocrystals become smaller steadily. On the other hand, owing to crystal surface energy differences and their distinct reactive activity toward sulfur, an increase in the amount of CS_2_ promotes vertical growth of anisotropic crystal (Mansouri and Semagina, 2018), and the thickness of nanosheets increases.

The assembly or aggregation of layered nanosheets can be influenced by the surface passivation and polarity of solvent, owing to the combined effect of solvation and cohesive energy (Zhang X. et al., 2017). The NbS_2_ nanosheets tend to assemble or aggregate with the CS_2_ amount increase, the possible reason is that more CS_2_ or H_2_S molecules adsorb to the surface of NbS_2_ nanosheets in the reaction solution, and occupy the sites of OLA molecules, thus the surface energy of NbS_2_ increases, and an assemble or aggregate phenomenon can more easily to occur.

### Electrochemical Performance of NbS_2_ Nanosheets

**Figure 5:**
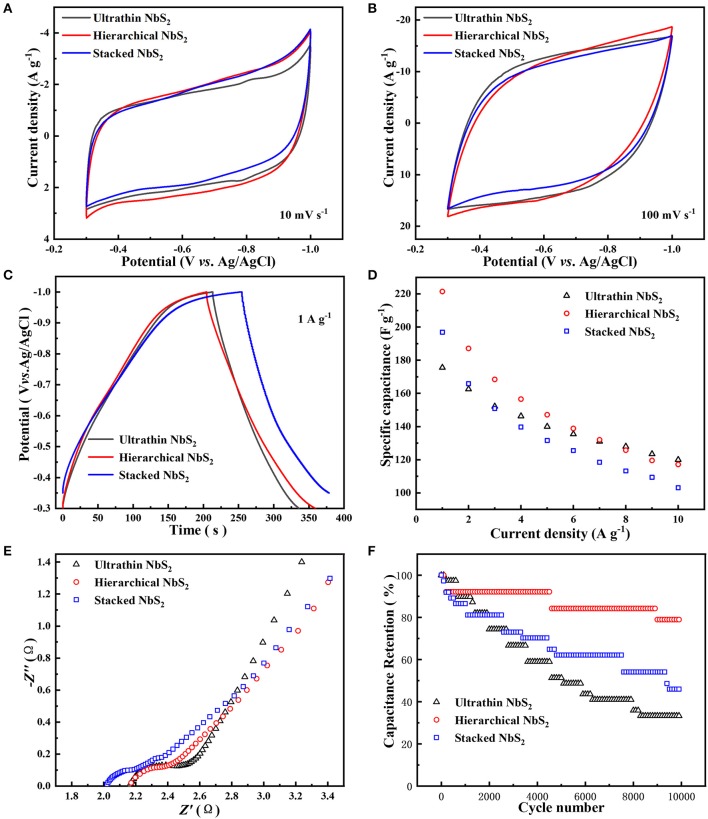
Electrochemical performance of ultrathin, hierarchical and stacked NbS_2_ nanosheets toward supercapacitor using a three-electrode electrolytic cell in neutral 1 M KCl aqueous solution. **(A)** CV curves at 10 mV s^−1^. **(B)** CV curves at 100 mV s^−1^. **(C)** GCD curves at current density of 1 A g^−1^. **(D)** Specific capacitance at different current densities. **(E)** EIS spectrum. **(F)** Cycling stability at 10 A g^−1^ for 10,000 cycles.

To compare the electrochemical performance differences of NbS_2_ nanosheets with diverse morphologies, the electrochemical tests of ultrathin, hierarchical, and stacked NbS_2_ nanosheets were investigated as negative electrode materials for a supercapacitor using a three-electrode electrolytic cell in neutral 1 M KCl aqueous solution (Figure 5). The CV curves of three samples all show quasi-rectangular shapes at scan rates of 10 and 100 mV s^−1^ (Figures 5A,B). There existed weak redox peaks at −0.82 V and −0.78 V (vs. Ag/AgCl) for the ultrathin sample at scan rate of 10 mV s^−1^, which may be due to faster ion diffusion than the other two samples. The operating voltage windows of galvanostatic charge-discharge (GCD) curves was −0.3 ~ −1V (vs. Ag/AgCl) for ultrathin and hierarchical NbS_2_ electrodes, and −0.35 ~ −1V (vs. Ag/AgCl) for stacked NbS_2_ electrodes because its discharge process was sluggish as the potential came near to −0.3 V (vs. Ag/AgCl) at the current density of 1 A g^−1^ (Figure 5C). The GCD curves of the ultrathin and hierarchical NbS_2_ electrodes performed a good symmetric triangular shape at 1 A g^−1^, suggesting its rapid and reversible charge-discharge ability (Liu et al., 2018a; Li et al., 2019), while the stacked NbS_2_ electrode showed slow charge process when the potential approached to −1.0 V (vs. Ag/AgCl) (Figure 5C). The specific capacitance can be calculated using Equation (S1) or Equation (S2) from Supplementary Material. The ultrathin NbS_2_ electrode had the highest rate capability, and displayed 120.0 F g^−1^ at 10 A g^−1^. However, the hierarchical NbS_2_ electrode showed the highest specific capacitance at low current densities (1 to 7 A g^−1^), displaying 221.4 F g^−1^ at 1 A g^−1^ (Figure 5D). The electrochemical energy storage performance of the stacked NbS_2_ electrode was poorer than the former two samples at high current densities.

To estimate the ion accessible surface area of NbS_2_ nanosheets electrodes, the electrochemical surface area (ECSA) was evaluated using cyclic voltammetry measurement (Figure S3). The electrochemical double layer capacitance (C_EDL_) of the ultrathin, hierarchical, and stacked NbS_2_ electrodes were 206.6, 195.8, and 165.5 mF respectively. The C_EDL_ of the ultrathin NbS_2_ electrode was the highest among three samples, while its specific capacitance was the lowest in low current densities (1 and 2 A g^−1^), demonstrating the ECSA was not the only affected factor for the performance of specific capacitance. The ECSA may play a more important role in the rapid charge and discharge process as the specific capacitance of ultrathin NbS_2_ electrodes was the highest in high current densities (8, 9, and 10 A g^−1^).

The EIS test was carried out to understand the internal resistance of the various NbS_2_ electrodes and interactions of the electrode/electrolyte interface. As shown in Figure 5E, the Nyquist plots displayed semicircles in the high frequency region, relating to charge transfer resistance (*R*_ct_) (Liu et al., 2018b). The *R*_ct_ value of the hierarchical NbS_2_ electrode was smaller than the ultrathin one, reflecting the easier process of charge transfer, which was coherent with the results that the specific capacitance of the former electrode was higher than the latter one in low current densities (Figure 5D). The slope of the ultrathin NbS_2_ electrode was the steepest among the three samples in the low frequency region, indicating more efficient ion diffusion, which was consistent with the higher rate capability shown in Figure 5D.

To test the potential application of NbS_2_ in practical uses, the cycling stability test was carried out at current density of 10 A g^−1^. The hierarchical NbS_2_ electrode exhibited capacitance retention with 78.9% of the initial specific capacitance after 10 000 cycles—much better than the ultrathin and stacked NbS_2_ electrodes (Figure 5F). The structure of the hierarchical NbS_2_ sample maintained well after the stability test, indicating that decay of capacitance may mainly be due to the exfoliation of partial active materials from the electrode (Figure S4). However, both the ultrathin and stacked NbS_2_ nanosheets aggregated to some extent, consistent with poor stability.

**Figure 6:**
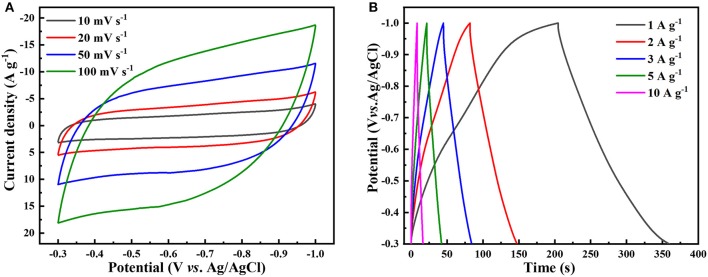
Capacitive performance of hierarchical NbS_2_ nanosheets toward supercapacitor using a three-electrode electrolytic cell in neutral 1 M KCl aqueous solution. **(A)** CV curves at different scan rates. **(B)** GCD curves at different current density.

As the hierarchical NbS_2_ electrode showed better overall electrochemical performance than the ultrathin or stacked NbS_2_ electrode, and the capacitive characteristics of the three electrodes are similar, we only discuss the capacitive performance of the hierarchical NbS_2_ electrode in more detail here. The capacitive performance of ultrathin and stacked NbS_2_ electrodes can be obtained from supporting information (Figure S6). Figure 6A shows the CV curves of the hierarchical NbS_2_ electrode tested at scan rates from 10 to 100 mV s^−1^ in the potential range of −0.3 ~ −1V (vs. Ag/AgCl), which displayed quasi-rectangular shapes, indicating the electrochemical double layer capacitance (EDLC) mechanism primarily (Peng et al., 2019). The hierarchical NbS_2_ electrode material occasioned oxidation reaction when the operating voltage window was broadened to −0.2 ~ −1 V and −0.1 ~ −1V (vs. Ag/AgCl), as the CV curves at 50 mV s^−1^ shows (Figure S5).

The GCD curves of NbS_2_ electrodes indicated the capacitive behavior at different current densities. The near-triangular GCD curves of the hierarchical NbS_2_ recorded from 1 to 10 A g^−1^ are shown in Figure 6B, exhibiting good specific capacitance of 221.4 F g^−1^ at 1 A g^−1^ and 117.1 F g^−1^ at 10 A g^−1^, respectively. The CV curves and the GCD curves of the ultrathin and stacked NbS_2_ are presented in Figure S6. The specific capacitance of ultrathin NbS_2_ was 175.6 F g^−1^ at 1 A g^−1^ and 120.0 F g^−1^ at 10 A g^−1^, respectively. And the specific capacitance of stacked NbS_2_ was 196.9 F g^−1^ at 1 A g^−1^ and 103.1 F g^−1^ at 10 A g^−1^, respectively.

From the electrochemical analysis, the hierarchical NbS_2_ electrode showed great overall electrochemical performance for energy storage. Fast and efficient electron transfer of the electrode can be achieved owing to the small charge transfer resistance. Furthermore, the restacking or aggregation of nanosheets can be suppressed by constructing a hierarchical structure, and the electrochemical active materials can be fully utilized during long-term charging and discharging processes.

## Conclusions

In summary, we introduce a colloidal synthesis method to regulate the morphologies of colloidal NbS_2_ nanocrystals via dynamics control. The size, thickness, and structure of nanocrystals can be adjusted by controlling the growth temperature and CS_2_ amount. The ultrathin NbS_2_ nanosheets show more efficient ion diffusion in the application of supercapacitor, while the self-assembled hierarchical structure NbS_2_ can suppress the restacking or aggregation of nanosheets effectively. This work offers insight into morphology regulation of layered TMDs in the synthesis process. The self-assembling mechanism has not yet been fully understood and needs to be investigated in future research.

## Data Availability Statement

All datasets generated for this study are included in the article/Supplementary Material.

## Author Contributions

WL (Investigation: Lead, Methodology: Lead, Writing – original draft: Lead, Writing – review and editing: Lead). XW (Investigation: Supporting, Writing – review and editing: Supporting). HD and YO (Writing – review and editing: Supporting). SX and YY (Software: Supporting). PX and YZ (Supervision: Lead, Writing – review and editing: Lead).

### Conflict of Interest

The authors declare that the research was conducted in the absence of any commercial or financial relationships that could be construed as a potential conflict of interest.
