# Supplementary material for: Colloidal Synthesis of NbS2 Nanosheets: From Large-Area Ultrathin Nanosheets to Hierarchical Structures
Source: Front Chem. 2020 Apr 7;8:189. doi: 10.3389/fchem.2020.00189 (PMC7154151; doi:10.3389/fchem.2020.00189)
Supplement: Supplementary file 1 [file Data_Sheet_1.docx]

**Supplementary Material for**

**Colloidal synthesis of NbS_2_ Nanosheets:**

**from large-area ultrathin nanosheets to hierarchical structures**

*Wenhui Li^1^, Xijun Wei^1^, Hongmei Dong^1^, Yingqing Ou^1^,*

*Shenghuan Xiao^1^, Yang Yang^1^, Peng Xiao^2,*^, Yunhuai Zhang^1,*^*

^1^College of Chemistry and Chemical Engineering, Chongqing University, Chongqing, China

^2^College of Physics, Chongqing University, Chongqing, China

**Corresponding Authors**

*Tel. +86 2365678362

*E-mail: [xiaopeng@cqu.edu.cn](mailto:xiaopeng@cqu.edu.cn); [xp2031@163.com](mailto:xp2031@163.com)

**Notes**

The authors declare no competing financial interest.

**Calculation methods:**

For a three-electrode system, the specific capacitance (*C*_s_, F g^-1^) can be calculated from the CV area integral by using Eq. (S1) or from the GCD curve by using Eq. (S2) (Peng et al., 2019) :

$C_{S}=\frac{1}{mv(V_{p}-V_{n})}\int_{V_{n}}^{V_{p}} I\left( V \right)dV$ (S1)

$C_{S}=\frac{I\times\Delta t}{m\times\Delta V}$ (S2)

where *I*, *V*, *V*_p_, *V*_n_, *m*, *v*, Δ*t* and Δ*V* are the response current (A), the operating voltage potential of CV curves (V), the positive potential (V), negative potential (V), the mass of active materials (g), the scan rate (V s^-1^), the discharge time (s) and the operating voltage window of GCD curves (V), respectively.

**REFERENCES**

Peng, H., Yao, B., Wei, X., Liu, T., Kou, T., Xiao, P., et al. (2019). Pore and heteroatom engineered carbon foams for supercapacitors. Adv. Energy Mater. 9:1803665. doi: 10.1002/aenm.201803665


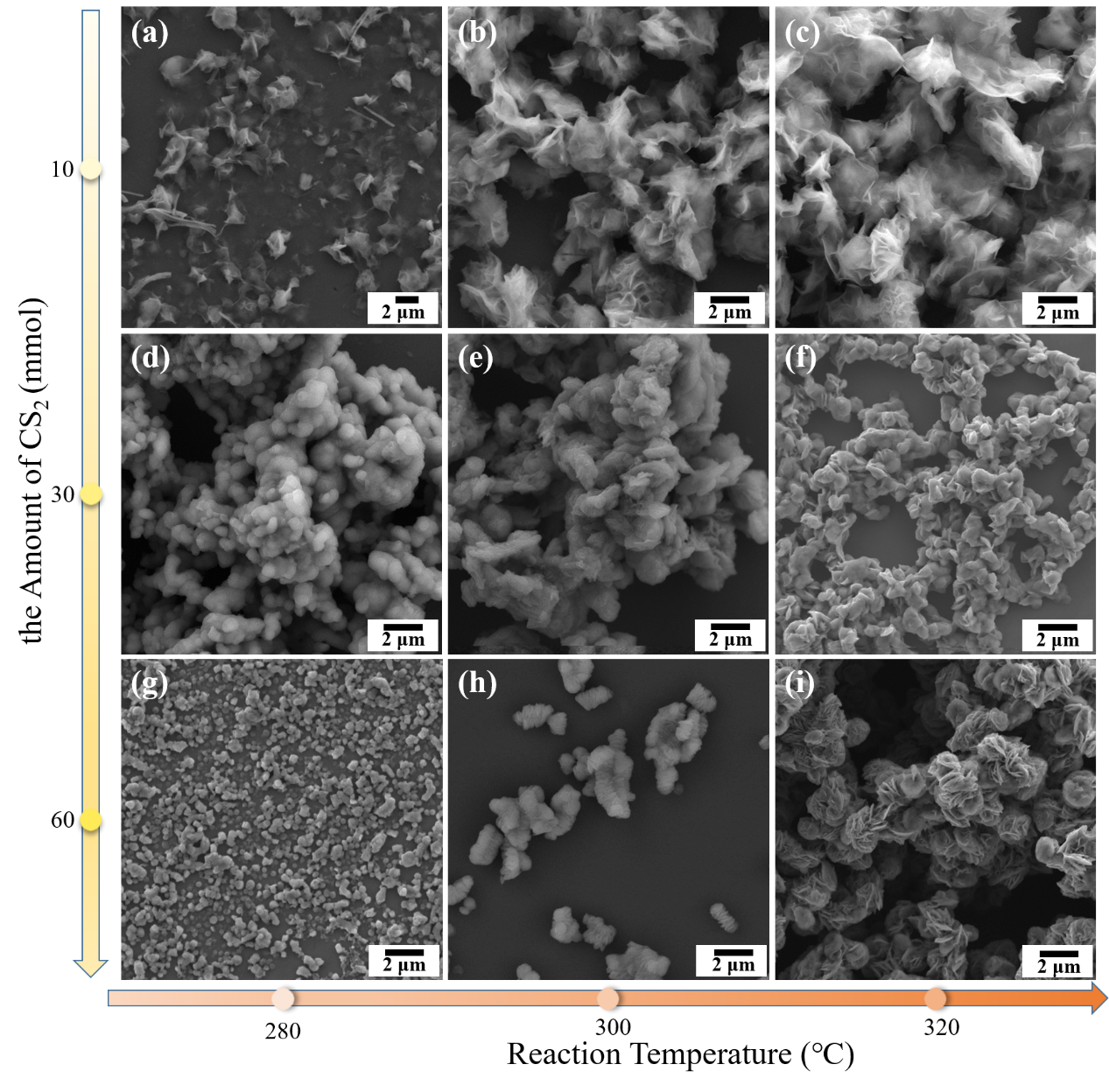


**Figure S1.** SEM images of NbS_2_ nanosheets synthesized at different reaction temperatures and CS_2_ amounts at low magnification. (a) 280 ℃, 10 mmol CS_2_. (b) 300 ℃, 10 mmol CS_2_. (c) 320 ℃, 10 mmol CS_2_. (d) 280 ℃, 30 mmol CS_2_. (e) 300 ℃, 30 mmol CS_2_. (f) 320 ℃, 30 mmol CS_2_. (g) 280 ℃, 60 mmol CS_2_. (h) 300 ℃, 60 mmol CS_2_. (i) 320 ℃, 60 mmol CS_2_.

**
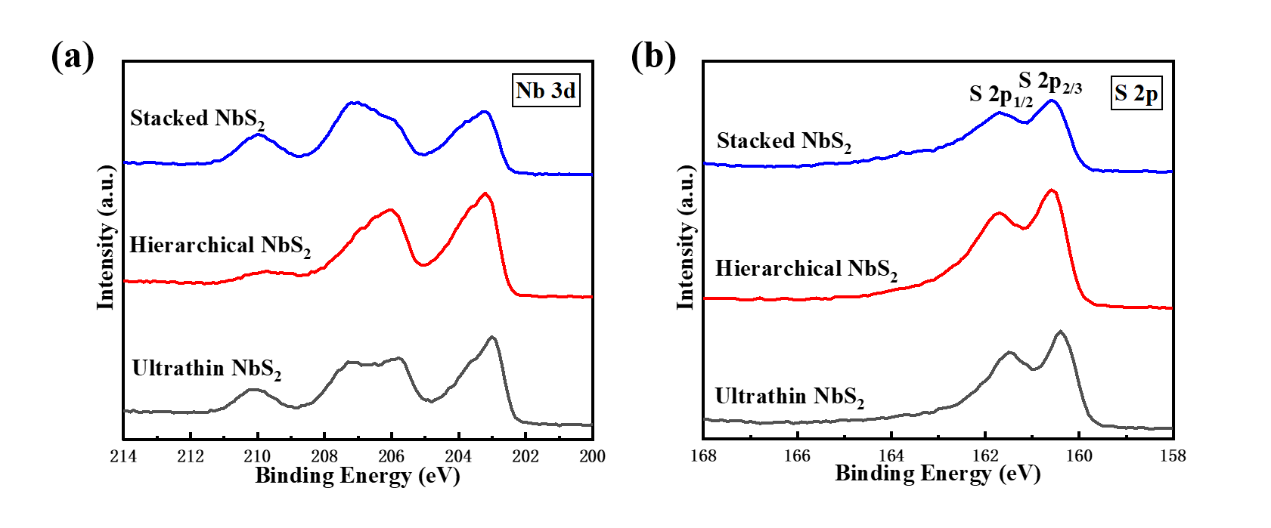
**

**Figure S2.** XPS results of ultrathin, hierarchical and stacked NbS2 nanosheets. (a) Nb 3d. (b) S 2p.

**
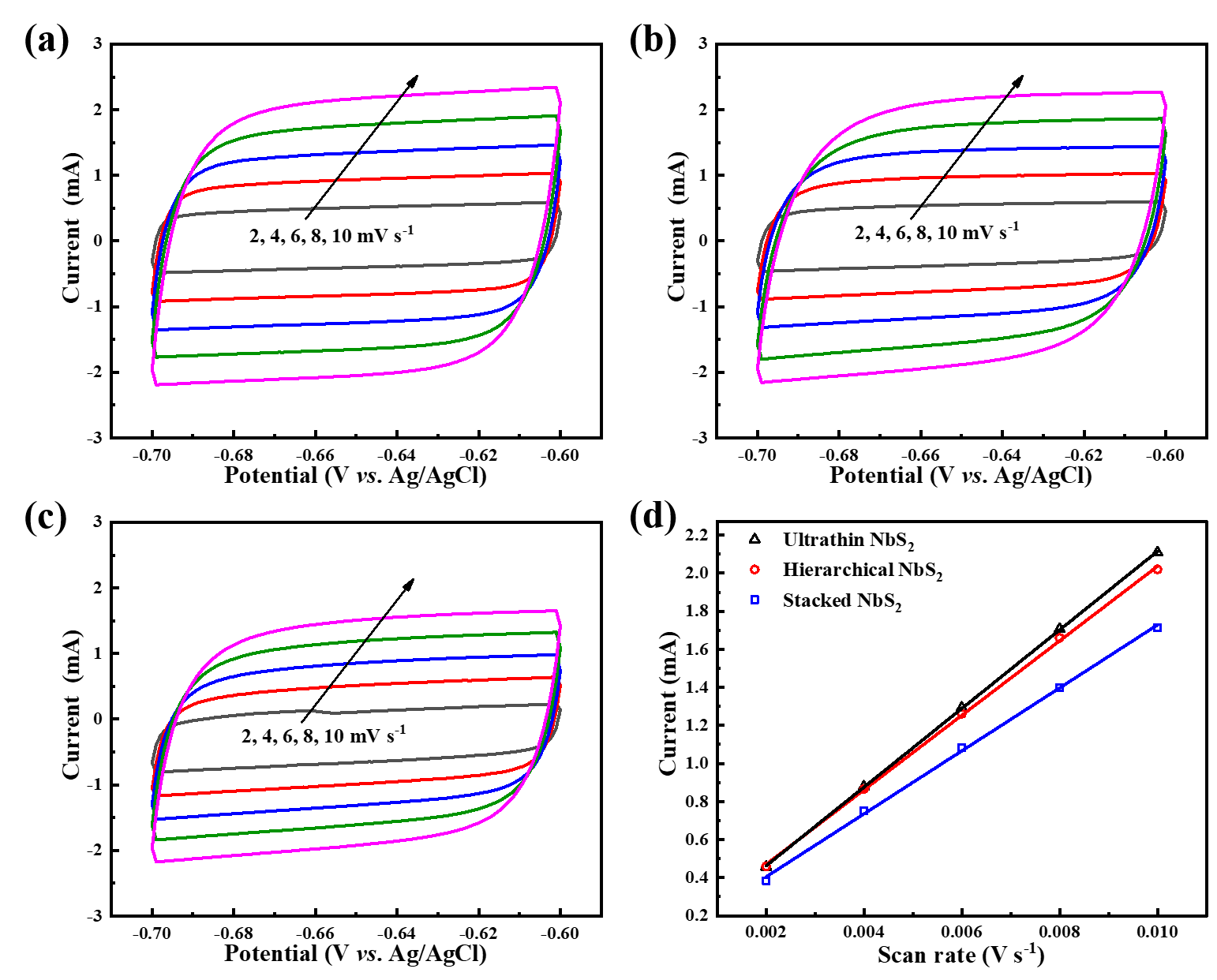
**

**Figure S3.** CV curves of (a) Ultrathin, (b) Hierarchical and (c) Stacked NbS_2_ electrode materials collected at different scan rates over a potential range of − 0.6 to − 0.7 V (*vs*. Ag/AgCl). (d) The I– ν plot of electrode materials at the potential of − 0.65 V (*vs*. Ag/AgCl) as a function of scan rates.

**
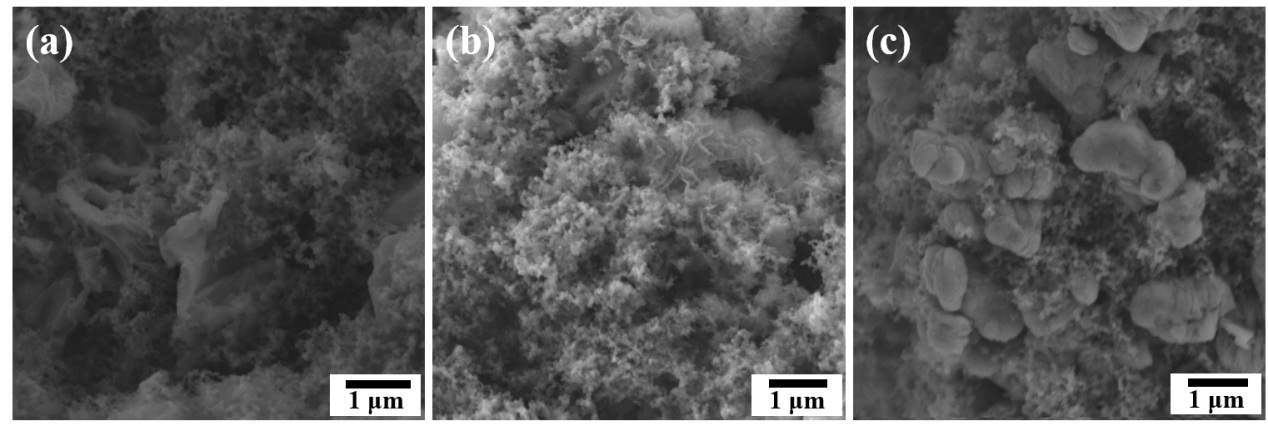
**

**Figure S4.** SEM images of electrode materials after stability test of 10 000 cycles for (a) Ultrathin, (b) Hierarchical and (c) Stacked NbS_2_ nanosheets.

**
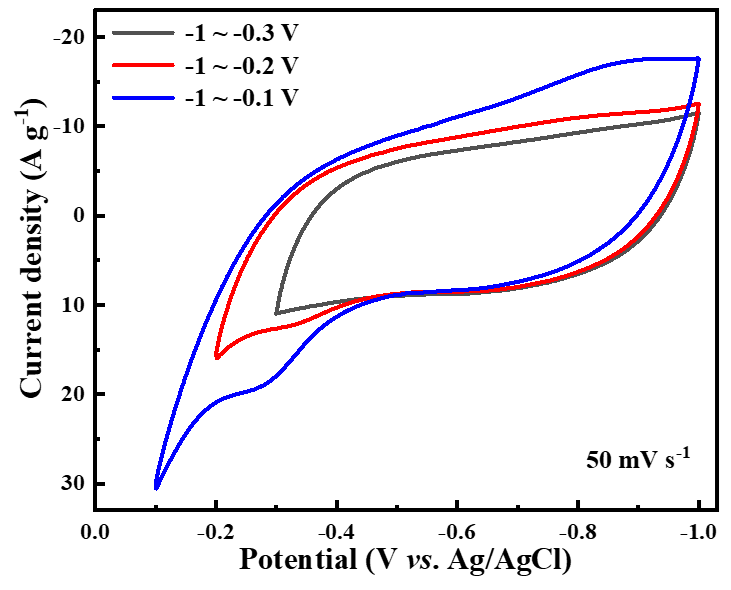
**

**Figure S5.** CV curves of hierarchical NbS_2_ nanosheets collected at different potential windows using a three-electrode electrolytic cell in neutral 1 M KCl aqueous solution.

**
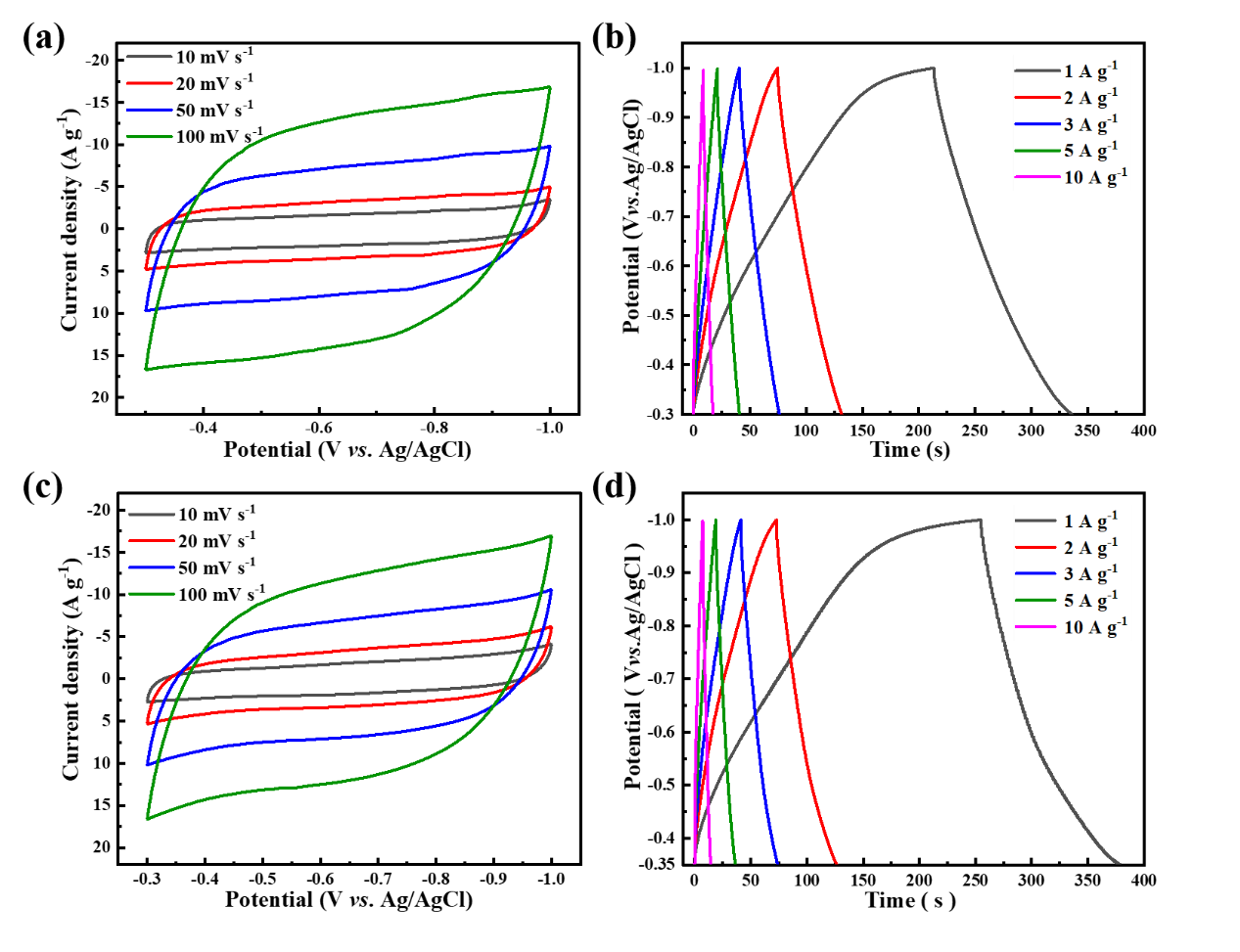
**

**Figure S6.** Capacitive performance of NbS_2_ electrodes toward supercapacitor using a three-electrode electrolytic cell in neutral 1 M KCl aqueous solution. (a) CV curves and (b) GCD curves of ultrathin NbS_2_ electrode. (c) CV curves and (d) GCD curves of stacked NbS_2_ electrode.
